# Children’s total blindness as a risk factor for early parent-child relationships: preliminary findings from an Italian sample

**DOI:** 10.3389/fpsyg.2023.1175675

**Published:** 2023-04-27

**Authors:** Anna Gui, Debora Perelli, Giulia Rizzo, Emilia Ferruzza, Elena Mercuriali

**Affiliations:** ^1^Centre for Brain and Cognitive Development, Birkbeck, University of London, London, United Kingdom; ^2^Robert Hollman Foundation, Padova, Italy; ^3^Department of Development and Socialization, University of Padova, Padova, Italy

**Keywords:** blindness, parental stress, social support, parent-child interaction, social engagement

## Abstract

**Background:**

Vision provides crucial information for parent-child attunement that scaffolds social development from the first months of life. Congenital blindness might affect both parental wellbeing and children’s behavior during parent-child interaction. In this study, we compared families of young children with total versus partial blindness to understand the link between residual vision, parenting stress and perceived social support, and children’s behavior during parent-child interaction.

**Methods:**

Participants were 42 white parents (21 fathers and 21 mothers) and their congenitally blind children (14 females, mean age = 14.81 months, SD = 10.46) with no co-occurring disability, recruited at the Robert Hollman Foundation rehabilitation centers in Italy. Parents’ scores on the Parenting Stress Index and Multidimensional Scale of Perceived Social Support questionnaires, as well as children’s behaviors signaling joint engagement during video-recorded episodes of parent-child interaction, were compared between the Total Blindness (TB, *n* = 12 children with no light perception or light perception in the dark but no quantifiable visual acuity) and Partial Blindness (PB, *n* = 9 children with a residual visual acuity below 3/60) groups.

**Results:**

We found that parents of TB children had higher parenting stress and lower perceived social support scores than parents of PB children. In fathers, total stress and stress linked to perceiving the child as difficult negatively correlated with perceived support from friends. There was no difference in the time TB and PB children spent displaying joint engagement behaviors during parent-child interaction. However, TB children directed their gaze and face less often toward their parents than PB children. We observed a trend of association between this behavior and maternal stress.

**Conclusion:**

These preliminary results suggest that the complete absence of vision from birth has adverse effects on stress linked to parenting and parental perceived social support. These findings support the importance of early family-centered interventions that extend to the parents’ communities and facilitate the parent-child dyad’s communication through non-visual behaviors. Replication is warranted in larger and more diverse samples.

## 1. Introduction

Sensory information guides the mutual adaptation between the newborn and their parents which is at the basis of the establishment of their relationships (Shultz et al., 2018). Vision is considered a crucial scaffolding function for social behavior, as it provides mutually reinforcing sensory inputs about the other person’s engagement in interaction (Johnson et al., 2015). In fact, as early as 10 min post-partum, newborns can direct their gaze toward the visual pattern of a face (Farroni et al., 2002). This facilitates early eye-contact between the child and their parents, that has cascading effects on the development of the social brain network (Senju and Johnson, 2009). Through vision, children gradually develop neurocognitive control mechanisms that respond to salient perceptual characteristics of the sensory world, learn to prioritize certain stimuli and select environmental inputs (Scerif, 2010). The neural circuits underlying these mechanisms determine visual attention skills and are also involved in the development of social attention (Klein et al., 2009). For example, by simply being exposed to the sensory features of faces, infants receive crucial information to develop complex social and communicative skills such as identity recognition, emotional expression recognition, speech sound learning and word acquisition, joint attention and engagement in communication (Carnevali et al., 2022).

The absence of information derived from the visual system especially during the maturation of the brain networks involved in social attention might have detrimental effects on the development of social skills (Tröster and Brambring, 1992). In line with this hypothesis, social and communication difficulties in the autism spectrum have higher prevalence in individuals with congenital blindness compared with the typical population (see for example Ek et al., 2005; Fink and Borchert, 2011; Jure et al., 2016; de Verdier et al., 2020). Early interventions directed at families of infants with a visual impairment have highlighted the importance of focusing on interaction, intersubjectivity and joint attention to support parent-child relationships (van den Broek et al., 2017). However, such interventions will only be successful if they precisely target the factors involved in the parent-child relationship that are most affected by the visual impairment. Understanding to what degree limitations in the information deriving from the visual system impact the family, and what aspects of the relationship are affected the most, is crucial to designing effective interventions tailored to the family’s needs.

An early diagnosis of a severe visual impairment poses a risk to the initial bond between the newborn and their parents as the child’s functional impairment and increasing demands involved in caring for the child constitute decisive sources of parental stress (Troster, 2001). Parents of visually impaired children tend to report higher anxiety and lower wellbeing than parents of typically developing children, although great variability is observed between families (Sola-Carmona et al., 2013). Interestingly, subjective wellbeing in parents of children with a visual impairment is linked to whether they feel their leisure time is limited by having a child with a disability (Sola-Carmona et al., 2016). Indeed, support from family and friends is a crucial resource for parents of children with disabilities (Kelso et al., 2005; Nabors et al., 2013) and perceived social support is correlated with stress levels in mothers of children with blindness or low vision (Troster, 2001). Further, parents of visually impaired children do feel they are less involved in intellectual and cultural activities (Leyser et al., 1996). However, thus far it is not clear whether higher stress levels and reduced wellbeing in the parents depend on the degree of their children’s sensory disability. Most studies included children with different degrees of visual impairment and co-occurring disabilities, and wide age ranges. This makes it difficult to disentangle whether parental stress and wellbeing in the early years are linked to the visual impairment alone or whether they are largely due to the presence of other comorbidities (Lupón et al., 2018). Further, no studies to our knowledge examined whether parental psychological wellbeing was different in families of children who have a residual vision compared to families of totally blind children.

The lack of visual information since infancy is also related to challenges experienced by parents in responding to their children’s cues when interacting with them (Campbell and Johnston, 2009). Congenital visual impairment is often linked to atypical developmental trajectories especially in social interaction skills, with possible setbacks between the second and the third year of life in blind children (Vervloed et al., 2020). Moreover, the communicative and expressive repertoire of visually impaired children is limited compared to sighted children during early parent-child interactions (Grumi et al., 2021). For example, with respect to sighted children, children with a severe visual impairment do not smile as frequently (Fraiberg, 1968, 1975). They present atypical or stereotyped movements not only when they are left alone, but also during episodes of play when experiencing excitement, frustration, or boredom (Fazzi et al., 1999). In the first 2 years of life, visually impaired children perform fewer social initiations in mother-child interactions during free play and respond to their mother’s initiations with fewer positive and more negative vocalizations (Rogers and Puchalski, 1984). Moreover, there is often a delay in the acquisition of language and perspective taking, which might disrupt parent-child attunement (Andersen et al., 1984; Brambring and Asbrock, 2010). Importantly, a recent study using a protocol for evaluating coordinated joint engagement in visually impaired children and their parents indicated that signs of engagement in the children were correlated with their visual acuity, emphasizing the need for further investigation on the role of residual vision in social development (Urqueta Alfaro et al., 2021).

In sum, raising a child with a visual impairment can be stressful for parents and could make them feel unsuitable for their role, with negative repercussions on the establishment of a positive relationship with the child in the first years of life (Troster, 2001). On the one hand, this might be linked to the challenge of having a child with a sensory disability, as indicated by studies reporting higher levels of stress and lower perceived support in parents of children with visual impairment compared to sighted children (Fathizadeh et al., 2012). On the other hand, the visual impairment might affect early parent-child interactions since children with low vision tend to show reduced reactivity to parental stimuli and fewer interactive initiations (Grumi et al., 2021). This might make it difficult for parents to interpret the child’s behavior, understand their needs and engage in social exchanges with them (Preisler, 1991; Tröster and Brambring, 1992; Baird et al., 1997). If congenital visual impairment *per se* constitutes a risk factor for parental burden and children’s engagement in social exchanges, these aspects would be exacerbated in families of children with total blindness, compared with families of children with a residual visual acuity. However, thus far the literature on social development in children with visual impairment has failed to address whether limited access to visual information alone has effects on early parent-child relationships. In fact, studies on legally blind children typically comprise both children with total blindness and children with a residual visual acuity.

The present research aimed to evaluate how the complete absence of vision from birth constitutes a risk factor for early parent-child relationships. To do this, we compared parental stress, perceived support, children’s signs of join engagement during parent-child interactions and the relationship between these factors between families of young children with total blindness (with and without light perception) with families of young children with low vision who had a residual visual acuity (i.e., partial blindness). Differently from previous studies (Lupón et al., 2018), we only included families of children with a congenital visual impairment but no other co-occurring disabilities or certified conditions, to tackle the role of vision on the development of early parent-child bonds. Further, while the literature thus far has predominantly examined these factors in relation to mothers (see Grumi et al., 2021 for a review), in this study we incorporated information from both mothers and fathers in relation to their child. We hypothesized the absence of access to visual information about the world plays a detrimental role for the parent-child relationship. Therefore, we expected that: (i) parents of totally blind children would have higher stress scores and lower perceived social support scores than parents of partially blind children, (ii) totally blind children would spend less time in behaviors signaling joint engagement than partially blind children during parent-child interaction, and (iii) parents with higher levels of stress would have children who spent less time displaying joint engagement behaviors.

## 2. Materials and methods

### 2.1. Participants

We recruited families of children with a severe visual impairment who accessed the Robert Hollman Foundation (RHF) rehabilitation centers in Padua and Cannero Riviera (Italy) when their child was younger than 36 months of age. This age criterion was chosen to focus on early parent-child relationships within the visually impaired children’s age range (3–40 months) examined in the previous literature reviewed by Grumi et al. (2021). Families were included in the study if: the child was between 0 to 36 months at the time of enrolment in the research; the child had a diagnosis of congenital blindness (total or partial) defined by the 138/2001 Italian law as having resolution visual acuity equivalent to recognition visual acuity below 3/60, as per the definition of blindness by the International Classification of Diseases (ICD) 11, even after any optical correction (such as lenses) has been applied; the child’s visual impairment was present from birth; the child had no comorbid condition or disability. Children were excluded if they had other medical diagnoses or disabilities, if data and consent were not obtained by both parents, or if at least one parent had a diagnosis or presented symptoms of psychopathology as evaluated by expert clinical psychologists at the RHF (see Section “2.2.3. Parental measures”).

Twenty-six families were recruited for this study. For five of those, data and/or consent was obtained from only one parent, therefore they were excluded from the current analyses. The final sample for the study included twenty-one families, resulting in 42 parents (21 mothers and 21 fathers) and 21 children (14 females and 7 males). Of these, 12 were assigned to the total blindness (TB) group and nine to the partial blindness (PB) based on their visual acuity (see Section “2.2.1. Visual acuity”). Gestational age at birth ranged from 24 to 42 weeks, with four children born before 36 gestational weeks. The children’s visual impairment was due to the following eye diseases: Eye malformations (including anophthalmia, microphthalmia, coloboma and morning glory anomaly), Leber Congenital Amaurosis, Retinopathy of Prematurity, Oculocutaneous albinism. All adults self-identified as white. In terms of parental highest educational attainment, 6 parents (14%, 3 mothers, 3 fathers) completed the middle school, 21 (50%, 11 mothers, 10 fathers) achieved a high-school degree, 13 (31%, 6 mothers, 7 fathers) had a university degree, and 2 (5%, 1 mother, 1 father) had a post-graduate degree (Supplementary Table 1 reports this information by group). All children were followed at the RHF with global assessments (including visual, neurodevelopmental, and psychological assessment and school counseling) and follow-ups. Two children (one in the TB and one in the PB group) also had education and occupational therapy at the RHF.

Table 1 summarizes the sample characteristics for the whole sample and by blindness group. Groups did not differ significantly in the proportion of females, parental education above or below university degree nor any of the age variables reported here (see Supplementary Table 2).

**TABLE 1:** Sample characteristics.

		Total blindness (TB) group	Partial blindness (PB) group	Whole sample
		M (SD) [range]		
Chronological age (in months)		14.89 (10.28) [4–38]	14.69 (11.31) [4–33]	14.81 (10.33) [4–38]
Gestational age at birth (in weeks)		37.08 (6.54) [24–42]	37.00 (5.59) [24–42]	37.05 (5.93) [24–42]
Age when accessed RHF (in months)		10.45 (6.30) [3–24]	8.79 (7.76) [1–25]	9.74 (6.74) [1–25]
Time followed at RHF (in months)		4.44 (6.73) [0–24.86]	5.91 (7.95) [0–24.2]	5.07 (7.03) [0–24.86]
Number of siblings		0.67 (0.76) [0–2]	0.11 (0.32) [0–1]	0.43 (0.67) [0–2]
Reynell-Zinkin developmental age (in months)	Social adaptation	7.42 (3.73) [4–17.5]	8.94 (5.61) [3.0–18.0]	8.07 (4.57) [3–18.0]
	Sensorimotor understanding	5.83 (3.95) [2–14.0]	9.56 (4.94) [3.5–16.5]	7.43 4.68 [2–16.5]
	Exploration of environment	2.83 (3.64) [0.0–10.0]	4.11 (4.08) [0.0–10.0]	3.38 3.79 [0–10.0]
	Response to the sound and verbal comprehension	6.17 (4.61) [1–18.0]	8.22 (6.53) [2.0–24.0]	7.05 5.46 [1–24.0]
	Expressive language	4.75 (5.24) [1.0–18.0]	6.22 (5.56) [1.0–15.0]	5.38 5.30 [1–18.0]

M, mean; SD, standard deviation; RHF, Robert Hollman Foundation.

This project has been approved by the Ethical Committee for Psychological Research of the University of Padova (Protocol Number: 2333). Informed consent for themselves and their children was obtained from all the parents involved in the study.

### 2.2. Measures

#### 2.2.1. Visual acuity

All 21 children taking part in the research were classified as legally blind based on the Italian law 138/2001 and the ICD-11 definition of blindness (visual acuity below 3/60). Visual acuity was determined by an expert orthoptist blind to the scope of the study using the Teller Acuity Cards preferential looking test. The Teller preferential looking test is a behavioral test that relies on the child’s innate preference to look at visual patterns and is widely used to measure resolution visual acuity in children with visual impairment (Teller et al., 2005). A trained orthoptist administers it by presenting an opaque gray screen which shows simultaneously a 20 by 20 cm field comprised of black-and-white stripes (gratings) on one side and a 20 by 20 cm homogeneous gray field on the other side. The orthoplist, who is unaware of the black-and-white pattern’s location and presents the gray screen while looking at the child’s behavior from a viewing peephole, uses the child’s gaze direction or head turn to determine the gratings’ location.

The sample was divided in two groups based on the children’s residual visual acuity. The Total Blindness (TB) group included children with no light perception, children who could perceive the light presented in the dark but failed to orient to any of the Teller cards, and children who could orient toward the 0.23 cycles/cm Teller card (which corresponds to the “Low Vision card” to which an acuity equivalent is not given, Teller Acuity Cards II, 2014) but failed to orient to the 0.32 cycles/cm card. Children with a residual vision that allowed them to orient to the 0.32 cycles/cm card when presented at a minimum distance of 9.5 cm (equivalent to a Snellen acuity of 20/6400) and any other Teller card corresponding to a visual acuity equal or lower than 3/60 (Snellen acuity of 20/400) were assigned to the Partial Blindness (PB) group.

#### 2.2.2. Children’s psychomotor assessment

The children’s psychomotor development was measured using the Reynell-Zinkin Scales (Reynell and Zinkin, 1979), an instrument created to assess developmental level in blind children between 6 and 42 months (Vervloed et al., 2000). The scales were administered in one session at the RHF by expert clinicians who had met and interacted with the child the day before. The children involved in this study were assessed for the following scales: Social Adaptation, Sensorimotor Understanding, Exploration of Environment, Response to the Sound and Verbal Comprehension, Expressive Language. Developmental ages were derived from the children’s scores for each of these scales; the children’s developmental age for the two groups is reported on Table 1. There was no significant difference between the two groups for any of the scales (Supplementary Table 2).

#### 2.2.3. Parental measures

Our key instruments to measure parental wellbeing were the Italian versions of the Parenting Stress Index-Short Form (Abidin, 1997) and the Multidimensional Scale of Perceived Social Support (Zimet et al., 1988). The Parenting Stress Index–short form (PSI) consists in 36 items rated on a 5-point Likert scale (1 = “strongly agree” to 5 = “strongly disagree”) measuring self-reported stress associated to parenting (Abidin, 1997). The Italian version of the PSI presents good reliability and internal consistency (Guarino et al., 2008) and has been previously used to assess stress levels in mothers and fathers of infants (e.g., Vismara et al., 2016). Three subscale scores, namely Parental Distress, Parent-Child Dysfunctional Interaction, and Difficult Child, as well as a Total Stress score consisting in the composite score of these subscales, were derived from this questionnaire. Higher total and subscale scores in the PSI indicate higher levels of self-reported stress.

The Multidimensional Scale of Perceived Social Support (MSPSS) provides a subjective measure of social support adequacy (Zimet et al., 1988). It is composed by 12 items evaluated on a 7-point Likert scale ranging from 1 = “very strongly disagree” to 7 = “very strongly agree.” The Italian version of the MSPSS has good internal concurrent validity (Di Fabio and Busoni, 2008) and has been widely used to measure perceived social support in mothers of young children (e.g., Grumi et al., 2021). A Total Perceived Support score and scores for perceived support from the Family, Friends, and a Significant Other Person (each composed by four items) were obtained. Higher total and subscale scores of the MSPSS indicate higher perceived support.

A series of clinical instruments were used to exclude the presence of psychopathology in the parents. Those were: The Millon Clinical Multiaxial Inventory-III (Millon et al., 2006) to measure parents’ personality; The Family Adaptation and Cohesion Scale-III (Olson, 1986) to evaluate the couple wellbeing or difficulties; and the Parent Development Interview to measure the parents’ reflective functioning (Fonagy et al., 1998; Aber et al., 1999; Slade, 2005).

#### 2.2.4. Children’s engagement in parent-child interaction

Sessions of parents-child interaction were video-recorded at the RHF for 17 families (6 PB and 11 TB). Video-recordings with both parents and the child were not available for coding for the remaining four families. Both parents sat on a floor mat with their child, and they were instructed to play as they would do at home for around 20 min while being filmed. A basket of toys and multi-sensory objects was provided for them to use. The experimenters left the room.

Microanalytic coding of 1 s units, as recommended for behavioral coding of parent-infant interaction (Beebe et al., 2010), was performed on five uninterrupted minutes of videotaped parents-child play using ELAN (v6.3). The 5-min sequence started from 1 min after the onset of the video-recording, to allow the triad to settle and begin to use the provided toys and objects while ensuring the situation was still relatively novel and interesting for them (Wan et al., 2017). A 5-min duration was chosen in line with other studies that emphasized the usefulness of brief sequences to examine engagement and interaction initiations between children with and without a neurodevelopmental problem and their mothers and fathers (Sethna et al., 2019; Pijl et al., 2021).

One rater (third author) blind to the study hypotheses coded all the videos (*n* = 17). Seven of the videos (40%) were also coded by another independent rater (first author) to ensure coding reliability. Both raters were trained on the same protocol and were unaware of group status of the child before starting to code. We used a coding protocol recently developed by Urqueta Alfaro et al. (2021) to identify nine reliably observable behaviors signaling joint attention engagement in infants with visual impairment. These were: orienting the body toward the parent (body orientation), shifting the gaze or face toward the parent (gaze/face shift, of note this behavior does not require the child to make eye-contact and in the present study it was annotated whenever face shifts toward the parent were performed as well as gaze shifts), vocalizing in relation to the joint engagement (vocalization), encompassing emotional expressions including smiling (emotional expression), pointing toward or reaching the object of joint attention (pointing/reaching), interrupting movements to attend to the parent or object (pause motor), giving the object to the parent (give object), touching the parent (touch), listening (listen). Additionally, the “other” annotation was used to indicate additional child’s behaviors signaling engagement in social interaction (to be specified by the rater). As in Urqueta Alfaro et al. (2021), multiple behaviors could be annotated for each 1-s unit. We did not distinguish whether the child’s behavior was referred to the mother or the father as the social partner.

All videos were seen at least four times. The first time, the raters examined the video and annotated the children’s behaviors. The second time, they played the video-recordings again and checked if they missed or misclassified behaviors. The third time, they examined each annotated segment to ensure it referred to children’s behaviors reflecting their engagement in interaction. Specifically, as in Urqueta Alfaro et al. (2021), the raters evaluated if in the annotated segment: (a) the child was interested in the same object as the parent, (b) the parent acknowledged that the child was participating in the activity, and (c) the parent’s action influenced the child’s experience, engaging them with the object. If any of these three conditions was not met, the annotation was discarded. The fourth time, segments were checked for their exact start and end time, to make sure they were composed of 1-s units.

Following coding reliability checks, the “listen” and “other” annotations were excluded and we added a general annotation of “joint engagement” including all 1-s units where one or more of the remaining eight behaviors was annotated. Our key measures were the number of episodes children displayed each of the behaviors signaling joint engagement within the 5-min parents-child interaction session, and the proportion of time the child presented any of joint engagement behaviors (Urqueta Alfaro et al., 2021).

### 2.3. Statistical analyses

#### 2.3.1. Effect of blindness on parental stress and perceived support

The overall effect of blindness on parental perceived stress and support was tested using two multilevel linear mixed models (“lme” function of the “nlme” package in R, (Pinheiro et al., 2022). Each of the two baseline models included parents’ total score (Total Stress score from the PSI or Total Perceived Social Support scores from the MSPSS) as dependent variables and family as a random effect. The following variables were subsequently added to the model; the child’s age, time in months since they were followed at the RHF, the child’s gestational age, the child’s sex, the parent’s role (mother or father), the parent’s higher educational attainment, the blindness group (PB or TB). The last model included an interaction term between parental role and blindness group, to assess whether the effect of blindness on parental perceived stress and support was different for mothers and fathers. We included the time since children were receiving some sort of intervention at the RHF as a covariate because early interventions were previously associated with increased PSI child-related scores in mothers of visually impaired children (Troster, 2001) and suggested to increase joint engagement behaviors in the children (van den Broek et al., 2017). Children’s gestational age was included as some of the children (*n* = 4) were born preterm. Parental educational attainment was included as previous research has suggested that parental technical degree might have a marginal effect on measures of wellbeing in parents of blind children (Sola-Carmona et al., 2016). All subsequent models were evaluated, and significant improvement of fit of the updated models was tested using a chi-square likelihood ratio test.

To further investigate the effect of blindness on the perceived stress and support subdomain scores we used two multivariate analyses of variance (MANOVAs) with the subscales scores (Parental Distress, Parent-Child Dysfunctional Interaction, and Difficult Child for the PSI, Family, Friends, and Significant Other Person for the MSPSS, respectively) as multiple dependent variables. Blindness group, parental role and their interaction were added as independent variables, covarying for child’s age, time in months since they were followed at the RHF, the child’s gestational age, the child’s sex and the parent’s higher educational attainment. Significant results were followed-up with *post-hoc* ANOVAs testing the effect of the dependent variables on the single subscale scores. Eta-squared was used as a measure of the effect size.

To understand whether parental perceived stress and support were related, Pearson’s correlation between all the scores (PSI total, MSPSS total and three subscale scores for both the PSI and the MSPSS) were separately tested for mothers and fathers. *P*-values were adjusted for 28 multiple testings using False-Discovery Rate correction (Benjamini and Hochberg, 1995).

#### 2.3.2. Relationship between children’s engagement in parent-child interaction and blindness

Inter-rater coding reliability was assessed using intra-class correlation (ICC, Shrout and Fleiss, 1979) in 40% of the videos, in line with previous research focusing on quantitative measures of parent-child interaction (Sethna et al., 2019; Pijl et al., 2021). ICC of the mean duration and total number of annotations for the eight behaviors independently coded by the two raters was 0.91 and 0.92, respectively, indicating excellent reliability for these measures.

The number of episodes children participated in joint engagement for each behavior was compared between groups using ANOVA, with the child’s age, time in months since they were followed at the RHF, gestational age and sex as covariates. *P*-values corrected using FDR were reported to account for the eight simultaneous tests. We also asked if the total proportion of time annotated with at least one joint engagement behavior was affected by blindness group using ANOVA, including the same child’s variables as covariates.

Last, with two linear regressions, we tested whether maternal and paternal Total Stress were associated with the proportion of joint engagement behaviors during parents-child interaction. As an exploratory analysis to further investigate the finding on the effect of blindness on children’s behavior, we also tested the association between number of gaze/face shifts and maternal and paternal stress, respectively. The child’s age, time in months since they were followed at the RHF, the child’s gestational age, the child’s sex and the parent’s higher educational attainment were included as covariates in all the regression models.

## 3. Results

### 3.1. Effect of blindness on parental stress

**FIGURE 1:**
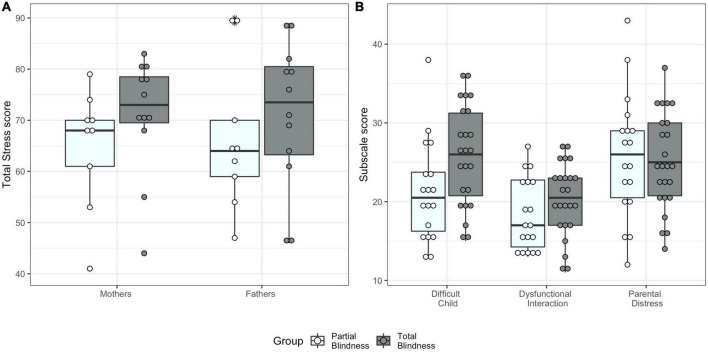
Parenting stress index scores by blindness group. **(A)** Total Stress score for mothers and fathers. **(B)** Parental scores for the three subscales (Difficult Child, Parent-Child Dysfunctional Interaction, and Parental Distress).

Parents of TB children showed on average higher Total Stress scores (*M*_*TB*_ = 71.08, SD_*TB*_ = 12.67) compared to parents of PB children [*M*_*PB*_ = 65.78, SD_*PB*_ = 12.80), χ^2^(12) = 6.61, *p* = 0.010]. This effect was not different between mothers and fathers [χ^2^(13) = 0.66, *p* = 0.415, Figure 1A]. The Supplementary Table 3 reports the results for all models.

Levene’s test indicated the assumption for homogeneity of variance for the three PSI subscales was met [Difficult Child: *F*(1,38) = 0.41, *p* = 0.751; Parent-Child Dysfunctional Interaction: *F*(1,38) = 0.46, *p* = 0.713; Parental Distress: *F*(1,38) = 0.07, *p* = 0.982]. Using Pillai’s trace, we found a significant effect of blindness group on the three parental stress subscale scores [*V* = 0.28, *F*(3,29) = 3.81, *p* = 0.020, η^2^ = 0.28, Figure 1B]. Specifically, we found that TB parents reported significantly higher Difficult Child scores compared to PB parents [*F*(1,31) = 10.07, *p* = 0.003, η^2^ = 0.25]. There was no significant effect of blindness on the other PSI subscale scores [Parent-Child Dysfunctional Interaction: *F*(1,31) = 2.59, *p* = 0.118, η^2^ = 0.08, Parental Distress: *F*(1,31) = 0.09, *p* = 0.772, η^2^ < 0.001, see Supplementary Table 4 for the other results].

### 3.2. Effect of blindness on perceived social support

**FIGURE 2:**
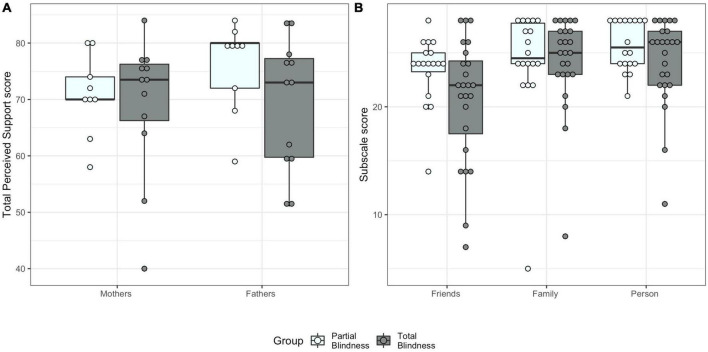
Multidimensional Scale of Perceived Social Support scores by blindness group. **(A)** Total Perceived Social Support score for mothers and fathers. **(B)** Scores for the three subscales (Support by Friends, Support by Family, Support by Significant Other Person).

Blindness group significantly explained Total Perceived Support scores [χ^2^(12) = 4.83, *p* = 0.028], with no differences between mothers and fathers [χ^2^(13) = 0.41, *p* = 0.521, Figure 2A]. Parents of TB children (*M*_*TB*_ = 69.08, SD_*TB*_ = 11.67) perceived less social support than parents of PB children (*M*_*PB*_ = 73.39, SD_*PB*_ = 7.88). Supplementary Table 5 reports all the models’ results.

Levene’s test confirmed homogeneity of variance for the three MSPSS subscales [Support by Family: *F*(3,38) = 0.36, *p* = 0.786; Support by Friends: *F*(3,38) = 1.86, *p* = 0.153; Support by Significant Other Person: *F*(3,38) = 0.60, *p* = 0.619]. Using Pillai’s trace, blindness group did not have a significant effect on the three subscale scores [*V* = 0.14, *F*(3,29) = 1.56, *p* = 0.220, η^2^ = 0.14, Figure 2B]. The other effects are reported in the Supplementary Table 6.

### 3.3. Relationship between parental stress and perceived support in mothers and fathers

As expected, mothers’ PSI Total Stress scores were significantly positively correlated with the PSI subscales (all *p*_*FDR*_ < 0.002). Maternal MSPSS Total Perceived Support scores were significantly positively correlated with the MSPSS subscale scores (all *p*_*FDR*_ < 0.001). Mothers’ perceived Support by Family and Support by Friends were also highly correlated (*r* = 0.75, *p*_*FDR*_ < 0.001). There was no significant correlation between PSI and MSPSS total and subscale scores (all *p*_*FDR*_ > 0.05).

**FIGURE 3:**
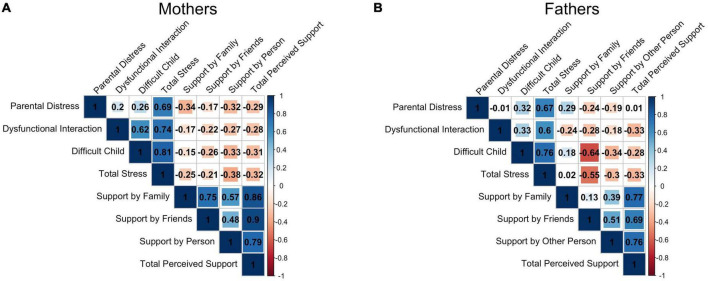
Correlation matrix between all subscale and total scores of the Parenting Stress Index (Parental Distress, Parent-Child Dysfunctional Interaction, Difficult Child, Total Stress) and Multidimensional Scale of Perceived Social Support (Support by Family, Support by Friends, Support by Other Significant Person and Total Perceived Support) for mothers **(A)** and fathers **(B)**.

For fathers too, PSI Total Stress scores were significantly positively correlated with the PSI subscale scores (all *p*_*FDR*_ < 0.015). Similarly, paternal MSPSS Total Perceived Support scores were significantly positively correlated with the MSPSS subscale scores (all *p*_*FDR*_ < 0.004). Interestingly, PSI Difficult Child scores were significantly negatively correlated with MSPSS Total score (*r* = −0.54, *p*_*FDR*_ = 0.040) and Support by Friends scores (*r* = −0.64, *p*_*FDR*_ = 0.008), indicating that fathers who felt less supported by friends were also more stressed and judged their children more difficult. Figure 3 represents these results.

### 3.4. Effect of blindness on children’s behavior in interaction

The proportion of time children displayed any type of joint engagement behaviors was not affected by blindness group [*F*(1,11) = 0.49, *p* = 0.499, η^2^ = 0.04, *M*_*TB*_ = 0.15, SD_*TB*_ = 0.10, *M*_*PB*_ = 0.19, SD_*PB*_ = 0.17]. Results testing the effect of blindness on the number of times children displayed each of the eight engagement behaviors are presented on Table 2. The only significant effect of blindness was found for the gaze/face shift behavior, annotated when the child shifted their gaze or face toward the parent. However, this result did not survive *p*-value correction for multiple testing (see Table 2).

**TABLE 2:** Summary statistics of the number of times children displayed engagement behaviors within the 5-min coded parents-child interaction session.

	Total blindness (TB) group, *n* = 11 M (SD)	Partial blindness (PB) group, *n* = 6 M (SD)	F(1,11)	*p*	*p*_*FDR*_	η^2^
Body orientation	1.09 (2.12)	0.83 (0.75)	0.08	0.782	0.893	0.007
Gaze/face shift	2.73 (2.15)	7.33 (4.63)	7.28	0.021*	0.168	0.40
Vocalization	5.82 (8.84)	7.33 (11.08)	1.06	0.324	0.865	0.09
Emotional expression	4 (4.15)	3.33 (3.67)	0.09	0.763	0.893	0.008
Pointing/reaching	6.27 (4.22)	6 (4.73)	0.02	0.893	0.893	0.002
Pause motor	2.36 (2.42)	1 (1.10)	1.36	0.268	0.865	0.11
Give object	0.46 (0.82)	0.667 (1.63)	0.37	0.555	0.893	0.03
Touch	0.91 (0.94)	0.667 (0.82)	0.27	0.612	0.893	0.02

M, mean; SD, standard deviation; *p* = *p*-value of the ANOVA, *p_FDR_* = *p*-value corrected for multiple testing using False Discovery Rate, η^2^ = eta-squared measure of the effect size.

*Indicates statistical significance at a threshold of *p* < 0.05.

### 3.5. Relationship between parental stress and children’s joint engagement

The relationship between the proportion of time children displayed joint engagement behaviors and parental Total Stress was not significant for both mothers (β = −0.004, SE = 0.004, *p* = 0.333) and fathers (β = 0.001, SE = 0.003, *p* = 0.830).

**FIGURE 4:**
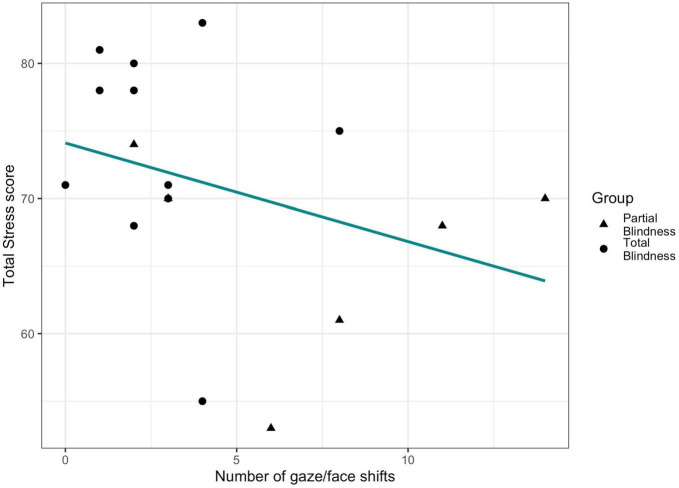
Relationship between maternal stress and children’s gaze/face shifts during the coded 5 min of parents-child interaction.

We observed a trend of association between number of gaze/face shifts displayed during the parents-child interaction and maternal Total Stress score (β = −0.25, SE = 0.16, *p* = 0.081, Figure 4). No significant relationship was found for fathers (β = 0.09, SE = 0.08, *p* = 0.336).

Since there was a significant difference between groups in the number of siblings (for all children, these were older siblings, see Supplementary Table 2), analyses were repeated with this variable as a covariate. Results confirmed the patterns reported above and can be found in Supplementary Tables 7–11.

## 4. Discussion

In this study we examined whether parental stress and perceived social support was different for parents of children with total compared to partial blindness, and whether this was linked to children’s behaviors signaling joint engagement during parent-child interaction. The analyses revealed that parents of totally blind children felt higher levels of stress linked to parenting and judged their child as more difficult, compared to parents of partially blind children. In the fathers, this feeling was associated to perceiving less support from friends. Overall, mothers and fathers of TB children also perceived less social support compared to parents of PB children, although there was no significant effect of blindness group for individual MSPSS subscale scores. We also observed that TB children performed gaze or face shift toward their parents less often than PB children during parent-child interaction. We found a trend of association between reduced number of children’s gaze/face shifts and increased levels of parenting stress in the mothers. Taken together, these findings suggest that a congenital absence of vision constitutes a risk factor for early parent-child relationships compared to having a severe visual impairment with residual vision.

Our findings indicate that knowing their children have access visual information (even if limited) changes the way parents perceive the child and how they feel supported by their social circle in their parenting role. The significant correlation between parenting stress and perceived support from friends in the fathers who participated in this study is in line with previous research on mothers of children with a visual impairment (Troster, 2001). The importance of vision in many aspects of Western societies causes stigmatization, disadvantage adults who are blind (Bulk et al., 2020) and could increase concern about the children’s future in parents (Troster, 2001). Families of totally blind children might feel particularly isolated because of this stigma. They are at risk of withdrawing from their social circles due to a combination of physical and mental overburden linked to medical care, concerns about the future and their children’s development, and experience of exclusion (Heiman, 2002; Baumgardner, 2019). The journey of parents from grief for a diagnosis of blindness to acceptance and empowerment can take up to 10 years (Cain and Fanshawe, 2021). In the meantime, increased parenting stress in the early years can have adverse effects on the parent-child attunement, with cascading effects on the social brain development (Azhari et al., 2019). For example, research on typically developing infants showed that the mother’s perception of infants’ sensory regulatory abilities is influenced by maternal stress linked to parenting (Quintigliano et al., 2021). The current research sustains the importance of early family-centered interventions that support the child development by understanding the parents’ needs and their community (Dempsey and Keen, 2008).

Examining measures from both parents allowed us to observe that the effects of the child’s complete absence of vision from birth on parental stress and perceived support were not different based on parental role. However, we found that parenting stress levels and perceiving the child as difficult were significantly associated to perceiving less support by friends in fathers only. Investigations on father-child interaction indicated that paternal sensitivity plays a role in shaping brain anatomy (Sethna et al., 2019) and cognitive functions (Towe-Goodman et al., 2014) in the first 3 years of age. Further, a secure father-child attachment as well as the involvement of fathers in early interventions have been linked to reduced risk of psychopathological outcome in children (Barker et al., 2017). Contrary to our predictions, we found that children’s behaviors signaling joint engagement was not associated with perceived stress linked to parenting. Although only marginally significant, we did observe a relationship between maternal stress levels linked to parenting and how often children directed their gaze or face toward the parent during episodes of parent-child interaction. Stress levels linked to parenting were higher in mothers of children who displayed less gaze or face shifts toward the parent when engaged in joint attention activities. This effect was not found in fathers. Taken together, these results suggest support to early parent-child relationship in totally and partially blind children should be addressed to both parents. For mothers, it might be particularly important to focus on helping them to interpret the signs of joint engagement of their children that are not dependent on vision. For fathers, it is crucial to feel supported by their social network and it might be more effective to help them understand how to convey their visually impaired child’s and family’s needs to their friends.

When examining parent-child interactions, we saw that, overall, children with total and partial blindness signal joint engagement in parent-child interaction to the same degree. Having or not a residual visual acuity also does not make a difference in the number of times behaviors are enacted, with one exception. Children who are partially blind tend to direct their gaze or face toward their parents more often during episodes of parent-child interaction, compared to totally blind children. We noticed a marginally significant association between this behavior and maternal perceived stress linked to parenting. Research in typically developing children indicates that mutual gaze increases neural synchronization between the infant and their mother (Leong et al., 2017). When children direct their gaze toward their parents during episodes of joint engagement, they give them feedback that increases alignment between the child and their parent, facilitating social engagement (Feldman, 2007). This important scaffolding mechanism is more impaired in totally blind children than in partially blind children, who seem to direct their gaze or face more often toward their interacting partner. A recent study showed that affective touch facilitates neural synchrony between sighted infants and mothers during face-to-face interactions (Nguyen et al., 2021). Future hyperscanning research should investigate whether non-visual modalities can completely substitute the role of mutual gaze on parent-child neural synchronization in totally blind children. This would provide further evidence for the importance of early interventions helping mothers to use the other joint engagement behaviors displayed by their totally blind children during interaction as feedback to compensate for the missed opportunities of parent-child attunement.

Carefully selecting our sample based on age range and diagnosis allowed us to have a cleaner design to examine the effects of complete congenital blindness on early parent-child relationships in the absence of parental psychopathology, but came at the expenses of the sample size. Indeed, a main limitation of this study is that the sample size is relatively modest, although similar or larger than most studies focusing on parent-child relationship in families of visually impaired individuals (see Grumi et al., 2021). Thus, a replication of the current results is warranted. The limited sample size had consequences on our findings and interpretations too. For example, TB children performed less gaze/face shifts toward their parents compared to PB children and it would have been interesting to investigate the association of this variable with parental stress separate by blindness group. However, our study was not powered enough to investigate this difference further. Larger samples would allow us to have more power for the analyses and to test mediation effects of children’s behaviors on the caregivers’ stress related to parenting.

Another limitation of the study is that our sample only included individuals living in Italy and self-identifying as white. Therefore, these findings should be interpreted in this specific cultural context and as such cannot be generalized. Studies conducted in non-Western countries indicate there too parents of children with visual impairment suffer high levels of stress. For example, research conducted in India reported high burden and depression felt by parents of infants and toddlers with congenital glaucoma (Dada et al., 2013). In China, parents of children with a visual impairment also shown higher levels of stress compared to parents of typically developing children (Lee et al., 2014). By increasing diversity in the study sample and conducting similar research in non-Western countries we would have the opportunity to evaluate the degree to which our results depend on the cultural characteristics of the sample (Garcini et al., 2022).

This research, if replicated, could have practical implications in clinical settings. It supports the importance of the role of practitioners who are expert about the communicative behaviors typically displayed by blind children (Fazzi et al., 1999) in supporting and promoting joint engagement between sighted parents and their children. Professionals who have experience and have been trained to work with children with a severe visual impairment would be in an optimal position to help parents to notice blind children’s signs of joint engagement that replace the gaze-related behaviors sighted parents tend to notice more easily. Preliminary findings on Video-feedback Intervention to promote Positive Parenting (VIPP) with visually-impaired children indicate early interventions should focus on promoting interaction, intersubjectivity and joint attention (van den Broek et al., 2017). However, these studies do not distinguish between children with total blindness and children who have a residual vision. Our research suggests that this is an important distinction; support to early parent-child relationships in the complete absence of the child’s vision should also primarily focus on parental wellbeing and the engagement of the family’s community. Increasing societal awareness about the implications of raising a child who is completely blind, and about the changes this introduces in the whole family’s life, might be crucial to increase parental availability toward their little one, and help them enjoy parenthood from early on.

## Data availability statement

The datasets presented in this article are not readily available because participants did not consent for their data to be shared. Requests to access the datasets should be directed to EM, e.mercuriali@fondazioneroberthollman.

## Ethics statement

The studies involving human participants were reviewed and approved by the Ethical Committee for Psychological Research of the University of Padova (Protocol Number: 2333). Written informed consent to participate in this study was provided by the participants’ legal guardian/next of kin.

## Author contributions

AG: conceptualization, methodology, formal analysis, validation, data curation, writing—original draft, and visualization. DP: conceptualization, methodology, investigation, resources, data curation, project administration, and writing—original draft. GR: validation and formal analysis. EF: conceptualization, methodology, investigation, writing—review and editing, supervision, and project administration. EM: conceptualization, methodology, investigation, resources, data curation, writing—original draft, supervision, project administration, and funding acquisition. All authors contributed to the article and approved the submitted version.
