# Supplementary material for: Children’s total blindness as a risk factor for early parent-child relationships: preliminary findings from an Italian sample
Source: Front Psychol. 2023 Apr 27;14:1175675. doi: 10.3389/fpsyg.2023.1175675 (PMC10172482; doi:10.3389/fpsyg.2023.1175675)
Supplement: Supplementary file 1 [file Data_Sheet_1.docx]

Supplementary Material

**Children’s total blindness as a risk factor for early parent-child relationships: Preliminary findings from an Italian sample**

**Supplementary Table S1.** Characteristics of the sample by blindness group.

|  | | Total Blindness (TB) group | Partial Blindness (PB) group |
| --- | --- | --- | --- |
| N children (%) | | 12 (57%) | 9 (43%) |
| N females (%) | | 8 (67%) | 6 (67%) |
| Diagnosis of visual impairment | *N (%) Eye malformations* | 6 (50%) | 2 (22%) |
|  | *N (%) Retinopathy of Prematurity* | 2 (17%) | 1 (11%) |
|  | *N (%) Leber Congenital Amaurosis* | 4 (33%) | 4 (45%) |
|  | *N (%) Oculocutaneous albinism* | 0 (0%) | 2 (22%) |
| N (%, N mothers/N fathers) | | | |
| Parent’s highest academic achievement | *Middle school degree* | 6 (14%, 3/3) | 0 (0%, 0/0) |
|  | *High-school degree* | 11 (26%, 6/5) | 10 (24%, 4/6) |
|  | *University degree* | 5 (12%, 2/3) | 8 (19%, 5/3) |
|  | *Post-graduate degree* | 2 (0%, 1/1) | 0 (0%, 0/0) |

**Supplementary Table S2.** Statistics testing the difference between the Total Blindness and Partial Blindness groups for the main characteristics of the sample reported on Supplementary Table S1 and on Table 1 of the main text.

|  | | Statistical test | statistics | P-value | Sign. |
| --- | --- | --- | --- | --- | --- |
| Child’s sex | | Fisher’s Exact test | OR=1 | 1 |  |
| Parent’s highest academic achievement (non-university vs university degree) | | Fisher’s Exact test | OR=0.52 | 0.347 |  |
| Chronological age (in months) | | t-test | t(16.40)=0.04 | 0.968 |  |
| Gestational age at birth (in weeks) | | t-test | t(18.60)=-0.03 | 0.975 |  |
| Age when accessed RHF (in months) | | t-test | t(15.16)=-0.53 | 0.606 |  |
| Time followed at RHF (in months) | | t-test | t(15.62)=0.45 | 0.660 |  |
| Number of siblings | | t.test | t(15.74)=-2.22 | 0.042 | * |
| Reynell-Zinkin Developmental age (in months) | *Social Adaptation* | t-test | t(13.12)=0.71 | 0.492 |  |
|  | *Sensorimotor Understanding* | t-test | t(15.00)=1.86 | 0.083 |  |
|  | *Exploration of Environment* | t-test | t(16.21)=0.74 | 0.468 |  |
|  | *Response to the Sound and Verbal Comprehension* | t-test | t(13.70)=0.81 | 0.434 |  |
|  | *Expressive Language* | t-test | t(16.79)=0.62 | 0.547 |  |

Abbreviations: OR = Odds Ratio, RHF = Robert Hollman Foundation.

**Supplementary Table S3.** Results of the multilevel mixed model with parental Total Stress score as dependent variable.

|  | *Model* | *df* | *AIC* | *BIC* | *logLik* | *Test* | *χ^2^* | *P-value* | *Sign.* |
| --- | --- | --- | --- | --- | --- | --- | --- | --- | --- |
| *Baseline model* | 1 | 3 | 334.81 | 340.02 | -164.41 |  |  |  |  |
| *Child’s age* | 2 | 4 | 334.47 | 341.42 | -163.23 | 1 vs 2 | 2.34 | 0.126 |  |
| *Time at RHF* | 3 | 5 | 333.00 | 341.69 | -161.50 | 2 vs 3 | 3.46 | 0.063 |  |
| *Child’s gestational age* | 4 | 6 | 334.98 | 345.41 | -161.49 | 3 vs 4 | 0.02 | 0.877 |  |
| *Child’s sex* | 5 | 7 | 336.67 | 348.83 | -161.33 | 4 vs 5 | 0.31 | 0.577 |  |
| *Parental role* | 6 | 8 | 338.62 | 352.52 | -161.31 | 5 vs 6 | 0.05 | 0.825 |  |
| *Parental education* | 7 | 11 | 336.59 | 355.70 | -157.29 | 6 vs 7 | 8.03 | 0.045 | * |
| *Blindness group* | 8 | 12 | 331.98 | 352.83 | -153.99 | 7 vs 8 | 6.61 | 0.010 | * |
| *Blindness X parental role* | 9 | 13 | 333.32 | 355.91 | -153.66 | 8 vs 8 | 0.66 | 0.415 |  |

Abbreviations: df = degrees of freedom, logLik = log likelihood, χ^2^= Likelihood ratio, sign. = significant at p<0.05, RHF = Robert Hollman Foundation.

**Supplementary Table S4.** Results of the MANOVA with Difficult Child, Parent-Child Dysfunctional Interaction and Parental Distress scores as dependent variables.

|  | *df* | *Pillai* | *Approx f* | *df1* | *df2* | *P-value* | *Sign.* |
| --- | --- | --- | --- | --- | --- | --- | --- |
| *Child’s age* | 1 | 0.31 | 4.38 | 3 | 29 | 0.012 | * |
| *Time at RHF* | 1 | 0.25 | 3.19 | 3 | 29 | 0.038 | * |
| *Child’s gestational age* | 1 | 0.20 | 2.41 | 3 | 29 | 0.087 |  |
| *Child’s sex* | 1 | 0.13 | 1.50 | 3 | 29 | 0.235 |  |
| *Parental role* | 1 | 0.07 | 0.77 | 3 | 29 | 0.521 |  |
| *Parental education* | 3 | 0.32 | 1.21 | 9 | 93 | 0.296 |  |
| *Blindness group* | 1 | 0.28 | 3.81 | 3 | 29 | 0.020 | * |
| *Blindness x parental role* | 1 | 0.04 | 0.45 | 3 | 29 | 0.722 |  |
| *Residuals* | 31 |  |  |  |  |  |  |

Abbreviations: df = degrees of freedom, sign. = significant at p<0.05, RHF = Robert Hollman Foundation.

**Supplementary Table S5.** Results of the multilevel mixed model with parental Total Perceived Support score as dependent variable.

|  | *Model* | *df* | *AIC* | *BIC* | *logLik* | *Test* | *χ^2^* | *P-value* | *Sign.* |
| --- | --- | --- | --- | --- | --- | --- | --- | --- | --- |
| *Baseline model* | 1 | 3 | 320.36 | 325.57 | -157.18 |  |  |  |  |
| *Child’s age* | 2 | 4 | 320.58 | 327.53 | -156.29 | 1 vs 2 | 1.78 | 0.182 |  |
| *Time at RHF* | 3 | 5 | 322.10 | 330.79 | -156.05 | 2 vs 3 | 0.47 | 0.491 |  |
| *Child’s gestational age* | 4 | 6 | 323.80 | 334.23 | -155.90 | 3 vs 4 | 0.30 | 0.585 |  |
| *Child’s sex* | 5 | 7 | 325.79 | 337.96 | -155.90 | 4 vs 5 | 0.01 | 0.918 |  |
| *Parental role* | 6 | 8 | 327.30 | 341.20 | -155.65 | 5 vs 6 | 0.49 | 0.482 |  |
| *Parental education* | 7 | 11 | 325.54 | 344.65 | -151.77 | 6 vs 7 | 7.76 | 0.051 |  |
| *Blindness group* | 8 | 12 | 322.71 | 343.56 | -149.35 | 7 vs 8 | 4.83 | 0.028 | * |
| *Blindness x parental role* | 9 | 13 | 324.29 | 346.88 | -149.15 | 8 vs 8 | 0.41 | 0.521 |  |

Abbreviations: df = degrees of freedom, logLik = log likelihood, χ^2^= Likelihood ratio, sign. = significant at p<0.05, RHF = Robert Hollman Foundation.

**Supplementary Table S6.** Results of the MANOVA with Support by Family, Support by Friends and Support by Significant Other Person as dependent variables.

|  | *df* | *Pillai* | *approx f* | *df1* | *df2* | *P-value* | *Sign.* |
| --- | --- | --- | --- | --- | --- | --- | --- |
| *Child’s age* | 1 | 0.07 | 0.75 | 3 | 29 | 0.529 |  |
| *Time at RHF* | 1 | 0.11 | 1.21 | 3 | 29 | 0.322 |  |
| *Child’s gestational age* | 1 | 0.05 | 0.53 | 3 | 29 | 0.665 |  |
| *Child’s sex* | 1 | 0.09 | 0.98 | 3 | 29 | 0.417 |  |
| *Parental Role* | 1 | 0.02 | 0.23 | 3 | 29 | 0.872 |  |
| *Parental education* | 3 | 0.37 | 1.43 | 9 | 93 | 0.185 |  |
| *Blindness group* | 1 | 0.14 | 1.56 | 3 | 29 | 0.221 |  |
| *Blindness x parental role* | 1 | 0.07 | 0.77 | 3 | 29 | 0.519 |  |
| *Residuals* | 31 |  |  |  |  |  |  |

Abbreviations: df = degrees of freedom, sign. = significant at p<0.05, RHF = Robert Hollman Foundation.

**Supplementary Table S7.** Results of the multilevel mixed model with parental Total Stress score as dependent variable when including a model covarying for the number of siblings.

|  | *Model* | *df* | *AIC* | *BIC* | *logLik* | *Test* | *χ^2^* | *P-value* | *Sign.* |
| --- | --- | --- | --- | --- | --- | --- | --- | --- | --- |
| *Baseline model* | 1 | 3 | 334.81 | 340.02 | -164.41 |  |  |  |  |
| *Child’s age* | 2 | 4 | 334.47 | 341.42 | -163.23 | 1 vs 2 | 2.34 | 0.126 |  |
| *Time at RHF* | 3 | 5 | 333.00 | 341.69 | -161.50 | 2 vs 3 | 3.46 | 0.063 |  |
| *Child’s gestational age* | 4 | 6 | 334.98 | 345.41 | -161.49 | 3 vs 4 | 0.02 | 0.877 |  |
| *Child’s sex* | 5 | 7 | 336.67 | 348.83 | -161.33 | 4 vs 5 | 0.31 | 0.577 |  |
| *Parental role* | 6 | 8 | 338.62 | 352.52 | -161.31 | 5 vs 6 | 0.05 | 0.825 |  |
| *Parental education* | 7 | 11 | 336.59 | 355.70 | -157.29 | 6 vs 7 | 8.03 | 0.045 | * |
| *Number of siblings* | 8 | 12 | 337.31 | 358.16 | -156.65 | 7 vs 8 | 1.28 | 0.258 |  |
| *Blindness group* | 9 | 13 | 333.93 | 356.52 | -153.97 | 8 vs 9 | 5.38 | 0.020 | * |
| *Blindness X parental role* | 10 | 14 | 335.23 | 359.55 | -153.61 | 9 vs 10 | 0.71 | 0.401 |  |

Abbreviations: df = degrees of freedom, logLik = log likelihood, χ^2^= Likelihood ratio, sign. = significant at p<0.05, RHF = Robert Hollman Foundation.

**Supplementary Table S8.** Results of the MANOVA with Difficult Child, Parent-Child Dysfunctional Interaction and Parental Distress scores as dependent variables, when adding number of siblings as covariate.

|  | *df* | *Pillai* | *Approx f* | *df1* | *df2* | *P-value* | *Sign.* |
| --- | --- | --- | --- | --- | --- | --- | --- |
| *Child’s age* | 1 | 0.32 | 4.35 | 3 | 28 | 0.012 | * |
| *Time at RHF* | 1 | 0.25 | 3.19 | 3 | 28 | 0.039 | * |
| *Child’s gestational age* | 1 | 0.20 | 2.38 | 3 | 28 | 0.091 |  |
| *Child’s sex* | 1 | 0.14 | 1.48 | 3 | 28 | 0.241 |  |
| *Parental role* | 1 | 0.07 | 0.75 | 3 | 28 | 0.534 |  |
| *Parental education* | 3 | 0.32 | 1.20 | 9 | 90 | 0.307 |  |
| *Number of siblings* | 1 | 0.04 | 0.38 | 3 | 28 | 0.771 |  |
| *Blindness group* | 1 | 0.29 | 3.82 | 3 | 28 | 0.021 | * |
| *Blindness x parental role* | 1 | 0.05 | 0.45 | 3 | 28 | 0.719 |  |
| *Residuals* | 30 |  |  |  |  |  |  |

Abbreviations: df = degrees of freedom, sign. = significant at p<0.05, RHF = Robert Hollman Foundation.

**Supplementary Table S9.** Results of the multilevel mixed model with parental Total Perceived Support score as dependent variable when including a model covarying for the number of siblings.

|  | *Model* | *df* | *AIC* | *BIC* | *logLik* | *Test* | *χ^2^* | *P-value* | *Sign.* |
| --- | --- | --- | --- | --- | --- | --- | --- | --- | --- |
| *Baseline model* | 1 | 3 | 320.36 | 325.57 | -157.18 |  |  |  |  |
| *Child’s age* | 2 | 4 | 320.58 | 327.53 | -156.29 | 1 vs 2 | 1.78 | 0.182 |  |
| *Time at RHF* | 3 | 5 | 322.10 | 330.79 | -156.05 | 2 vs 3 | 0.47 | 0.491 |  |
| *Child’s gestational age* | 4 | 6 | 323.80 | 334.23 | -155.90 | 3 vs 4 | 0.30 | 0.585 |  |
| *Child’s sex* | 5 | 7 | 325.79 | 337.96 | -155.90 | 4 vs 5 | 0.01 | 0.918 |  |
| *Parental role* | 6 | 8 | 327.30 | 341.20 | -155.65 | 5 vs 6 | 0.49 | 0.482 |  |
| *Parental education* | 7 | 11 | 325.54 | 344.65 | -151.77 | 6 vs 7 | 7.76 | 0.051 |  |
| *Number of siblings* | 8 | 12 | 326.18 | 347.03 | -151.09 | 7 vs 8 | 1.36 | 0.244 |  |
| *Blindness group* | 9 | 13 | 324.70 | 347.29 | -149.35 | 8 vs 9 | 3.48 | 0.062 |  |
| *Blindness x parental role* | 10 | 14 | 326.28 | 350.61 | -149.14 | 9 vs 10 | 0.42 | 0.518 |  |

Abbreviations: df = degrees of freedom, logLik = log likelihood, χ^2^= Likelihood ratio, sign. = significant at p<0.05, RHF = Robert Hollman Foundation.

**Supplementary Table S10.** Results of the MANOVA with Support by Family, Support by Friends and Support by Significant Other Person as dependent variables, when adding number of siblings as covariate.

|  | *df* | *Pillai* | *approx f* | *df1* | *df2* | *P-value* | *Sign.* |
| --- | --- | --- | --- | --- | --- | --- | --- |
| *Child’s age* | 1 | 0.07 | 0.73 | 3 | 28 | 0.544 |  |
| *Time at RHF* | 1 | 0.11 | 1.17 | 3 | 28 | 0.337 |  |
| *Child’s gestational age* | 1 | 0.05 | 0.51 | 3 | 28 | 0.678 |  |
| *Child’s sex* | 1 | 0.09 | 0.94 | 3 | 28 | 0.433 |  |
| *Parental Role* | 1 | 0.02 | 0.23 | 3 | 28 | 0.878 |  |
| *Parental education* | 3 | 0.37 | 1.39 | 9 | 90 | 0.205 |  |
| *Number of siblings* | 1 | 0.00 | 0.03 | 3 | 28 | 0.994 |  |
| *Blindness group* | 1 | 0.14 | 1.51 | 3 | 28 | 0.234 |  |
| *Blindness x parental role* | 1 | 0.07 | 0.74 | 3 | 28 | 0.540 |  |
| *Residuals* | 30 |  |  |  |  |  |  |

Abbreviations: df = degrees of freedom, sign. = significant at p<0.05, RHF = Robert Hollman Foundation.

**Supplementary Table S11.** Summary statistics of the number of times children displayed engagement behaviors within the 5-minutes coded interaction session, when adding number of siblings as covariate.

|  | F(1,10) | p | p_FDR_ | η^2^ |
| --- | --- | --- | --- | --- |
| Body orientation | 0.07 | 0.791 | 0.888 | 0.007 |
| Gaze/face shift | 6.69 | 0.027* | 0.216 | 0.40 |
| Vocalisation | 0.98 | 0.345 | 0.888 | 0.09 |
| Emotional expression | 0.10 | 0.758 | 0.888 | 0.008 |
| Pointing/reaching | 0.02 | 0.888 | 0.888 | 0.002 |
| Pause motor | 1.75 | 0.216 | 0.864 | 0.11 |
| Give object | 0.34 | 0.571 | 0.888 | 0.03 |
| Touch | 0.28 | 0.604 | 0.888 | 0.02 |

The proportion of time children displayed any type of joint engagement behaviors was not affected by blindness group (F(1,10)=0.50, p=0.499, η^2^=0.04) when adding number of siblings as a covariate in the ANOVA.

Similarly to the analyses reported in the main text, the relationship between the proportion of time children displayed joint engagement behaviors and parental Total Stress was not significant for both mothers (β=-0.003, SE=0.006, p=0.594) and fathers (β=0.0009, SE=0.003, p=0.792).

When adding the number of siblings as a covariate in the model, the association between number of gaze/face shifts displayed during the parents-child interaction and maternal Total Stress score was non-significant with no trend of association (β=-0.21, SE=0.17, p=0.251). Of note, number of siblings was not associated with number of gaze/face shifts (β=-0.68, SE=2.35, p=0.781). No significant relationship was found for fathers (β=0.08, SE=0.09, p=0.376), as in the main analyses.
